# Observation of Resistive Switching Memory by Reducing Device Size in a New Cr/CrO_*x*_/TiO_*x*_/TiN Structure

**DOI:** 10.1007/s40820-015-0055-3

**Published:** 2015-08-01

**Authors:** Debanjan Jana, Subhranu Samanta, Sourav Roy, Yu Feng Lin, Siddheswar Maikap

**Affiliations:** Thin Film Nano Tech. Lab., Department of Electronic Engineering, Chang Gung University, 259 Wen-Hwa 1st Rd., Kwei-Shan, Tao-Yuan, 333 Taiwan, ROC

**Keywords:** CrO_*x*_, TiO_*x*_, Resistive switching memory, Slope/shape factor, Device size

## Abstract

The resistive switching memory characteristics of 100 randomly measured devices were observed by reducing device size in a Cr/CrO_*x*_/TiO_*x*_/TiN structure for the first time. Transmission electron microscope image confirmed a via-hole size of 0.4 µm. A 3-nm-thick amorphous TiO_*x*_ with 4-nm-thick polycrystalline CrO_*x*_ layer was observed. A small 0.4-µm device shows reversible resistive switching at a current compliance of 300 µA as compared to other larger size devices (1–8 µm) owing to reduction of leakage current through the TiO_*x*_ layer. Good device-to-device uniformity with a yield of >85 % has been clarified by weibull distribution owing to higher slope/shape factor. The switching mechanism is based on oxygen vacancy migration from the CrO_*x*_ layer and filament formation/rupture in the TiO_*x*_ layer. Long read pulse endurance of >10^5^ cycles, good data retention of 6 h, and a program/erase speed of 1 µs pulse width have been obtained.

## Introduction

Recently resistive random access memory (RRAM) has been investigated for the next-generation non-volatile memory applications [1, 2]. The RRAM device offers a promise for NVM application due to its simple structure, low power consumption, high density, fast program/erase speed, and low cost [3]. Various materials like HfO_*x*_ [4], TaO_*x*_ [5, 6], TiO_*x*_ [7–10], and so on have been reported by many groups. Other materials such as ZnO [11], BaWO_4_ [12], and so on have been reported also. Among of them, TiO_*x*_ is one of the most promising materials owing to its fab-friendly, good thermal stability, adequate band gap (~3.0 eV) for low leakage, high dielectric constant (κ ~ 80), and so on [13]. Kwon et al. [14] have reported the bipolar resistive switching in Pt/TiO_2_/Pt structure at a high current compliance (CC) of 30 mA. Jeong et al. [15] have reported the bilayer switching layers in a Pt/Ni/a-TiO_2_/Al_2_O_3_/Pt structure with a CC of >1 mA. Park et al. [16] have unveiled the multi-bit resistive switching operation in an Ir/TiO_*x*_/TiN structure with a CC of 1 mA. Goren et al. [17] demonstrated bistable memory effect in Co/TiO_2_/TiO_*x*_/Co/Pd structure with a low CC of approximately 100 µA. Zeng et al. [18] have reported resistive switching characteristics using a Pt/ZrO_2_/TiO_2_/Pt structure at a CC of 10 mA. Strachan et al. [19] have reported the resistive switching characteristics using a Cr/Pt/TiO_2_/Pt structure at a CC of 250 µA.

Although many groups have reported these TiO_*x*_-based different RRAM devices, however, resistive switching characteristics by reducing device size as well as leakage current in a Cr/CrO_*x*_/TiO_*x*_/TiN structure have not been investigated yet. To obtain good resistive switching characteristics, both amorphous TiO_*x*_ switching and polycrystalline CrO_*x*_ oxygen vacancy supply layers are effectively combined with smaller device sizes. Microstructure and device size are confirmed by transmission electron microscope (TEM) image. The switching mechanism is owing to oxygen vacancy filament formation/rupture into the TiO_*x*_ switching layer. Weibull distribution plot of 100 randomly measured devices with a size of 0.4 × 0.4 µm^2^ shows good device-to-device uniformity with a yield of >85 %. It is found that higher slope/shape factor indicates higher uniformity of the devices. By investigating the scale factor, it is found that the device can be operated with low voltage of ±1 V and a low current of <300 µA. Long read pulse endurance of >10^5^ cycles, stable data retention of >6 h, and good program/erase (P/E) endurance with a pulse width of 1 µs are obtained, which indicate future application of this new Cr/CrO_*x*_/TiO_*x*_/TiN resistive switching memory device.

## Experimental

**Fig. 1:**
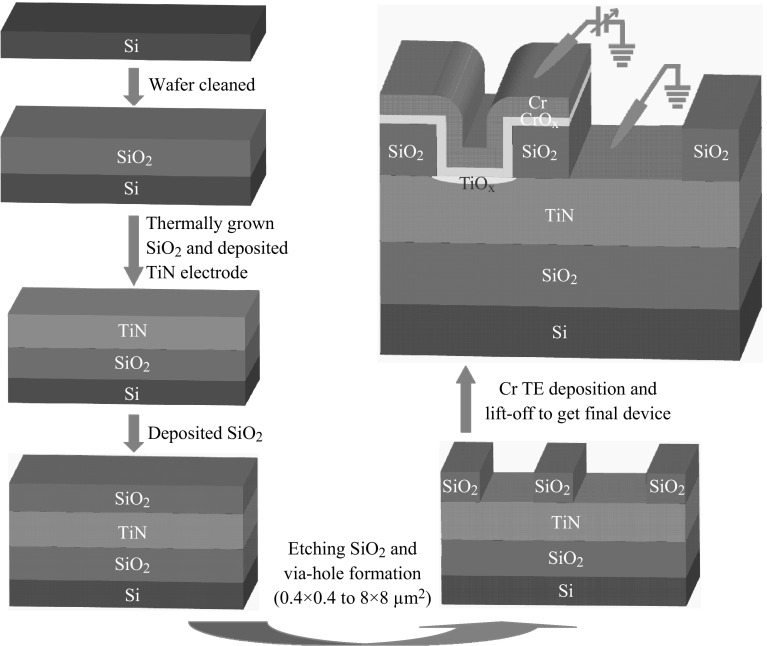
The RRAM device with sizes ranging from 0.4 × 0.4 to 8 × 8 µm^2^

The Cr/CrO_*x*_/TiO_*x*_/TiN RRAM devices were fabricated using 8-inch SiO_2_/Si wafers. The device process flow is shown in Fig. 1. The thickness of SiO_2_ layer was 200 nm. A 200-nm-thick titanium-nitride (TiN) as a bottom electrode (BE) was deposited on SiO_2_ layer. Next 150-nm-thick SiO_2_ layer was deposited to different pattern via-hole sizes ranging from 0.4 × 0.4 to 8 × 8 µm^2^ using both photolithography and dry etching processes. Then, 8 inch wafer was broken to 2 × 2 inch^2^ pieces and did next step. The chromium (Cr) metal as a top electrode (TE) was deposited by radio frequency (RF) sputtering. The Cr metal target with a constant argon (Ar) gas flow rate of ten sccm was used. During deposition, chamber pressure and deposition power were 6 mTorr and 100 W, respectively. A TiO_*x*_ resistive switching layer with a CrO_*x*_ layer was observed after deposition of Cr TE. Finally, lift-off process was done to get a simple Cr/CrO_*x*_/TiO_*x*_/TiN structure. More than 150 devices for each size were obtained on a small piece of 8 inch wafer.

The memory characteristics were investigated by measuring 100 randomly picked devices. Thickness and microscopic structure of Cr/CrO_*x*_/TiO_*x*_/TiN RRAM device were analyzed by transmission electron microscopy with energy of 200 keV. Electrical characteristics were measured by Agilent 4156C/B1500 precision semiconductor analyzer. During measurement, the bias was applied on the TE and the BE was grounded.

## Results and Discussion

**Fig. 2:**
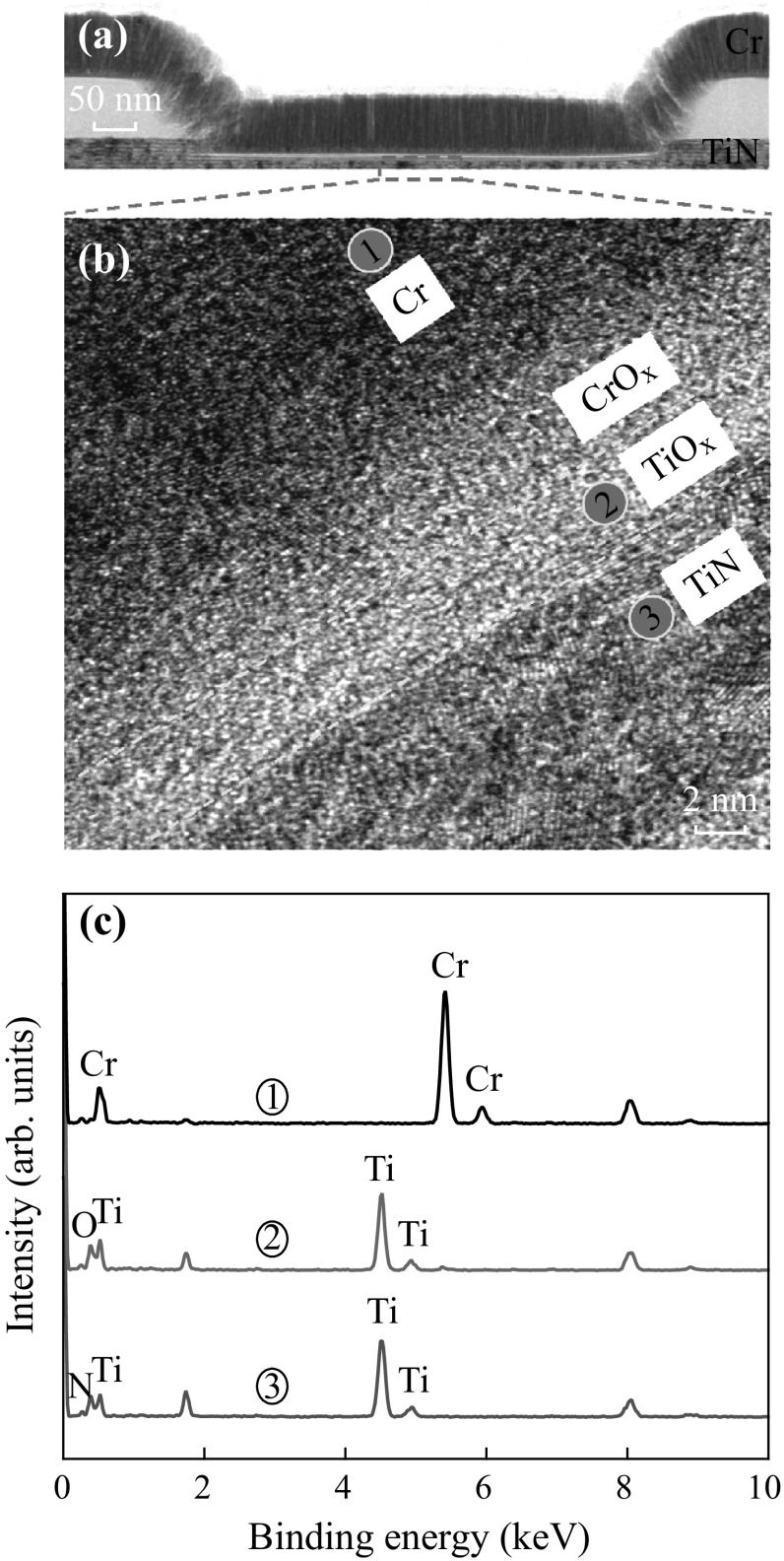
**a** TEM image of Cr/CrO_*x*_/TiO_*x*_/TiN RRAM device with a via-hole size of 0.4-µm device. **b** HRTEM image shows a CrO_*x*_ layer in between TiO_*x*_ switching and Cr electrode because of partially oxidization of Cr metal during deposition. **c** EDX spectrum confirms the presence of Cr, Ti, O, and N elements in* 1*,* 2*, and* 3* denoted layers of (**b**)

Figure 2a shows TEM image of a typical via-hole size of 0.4 × 0.4 µm^2^. The length of via-hole is found to be approximately 0.5 µm. A thickness of Cr layer is approximately 150 nm. High-resolution TEM image on inside via-hole region confirms the layer-by-layer of Cr/CrO_*x*_/TiO_*x*_/TiN structure (Fig. 2b). The thickness of amorphous TiO_*x*_ layer is approximately 3 nm, which acts as a switching layer. This TiO_*x*_ layer is grown during the Cr TE deposition owing to lower Gibbs free energy of TiO_2_ (−887.6 kJ (mol)^−1^ at 300 K). In addition, reactive Cr TE is also partially oxidized at the Cr/TiO_*x*_ interface and creates CrO_*x*_ owing to lower Gibbs free energy of Cr_2_O_3_ of −694.88 kJ (mol)^−1^ [20, 21]. This CrO_*x*_ layer with a thickness of approximately 4 nm is polycrystalline. Elemental analysis of Cr/CrO_*x*_/TiO_*x*_/TiN structure has been explored by EDX spectrum. The existence of Cr, Ti, O, N elements in corresponding layers of 1, 2, 3 are shown in Fig. 2c. The peaks’ position of Cr, Ti, O, N elements are found to be 5.4, 4.5, 0.4, and 0.28 keV, respectively. Due to both lower Gibbs free energy and deposition by sputtering, the defective CrO_*x*_ layer is formed or oxygen vacancy is observed in the CrO_*x*_ layer. Therefore, this CrO_*x*_ layer acted as an oxygen vacancy supply layer to form conductive filament into the TiO_*x*_ switching layer and good switching characteristics can be observed below.

**Fig. 3:**
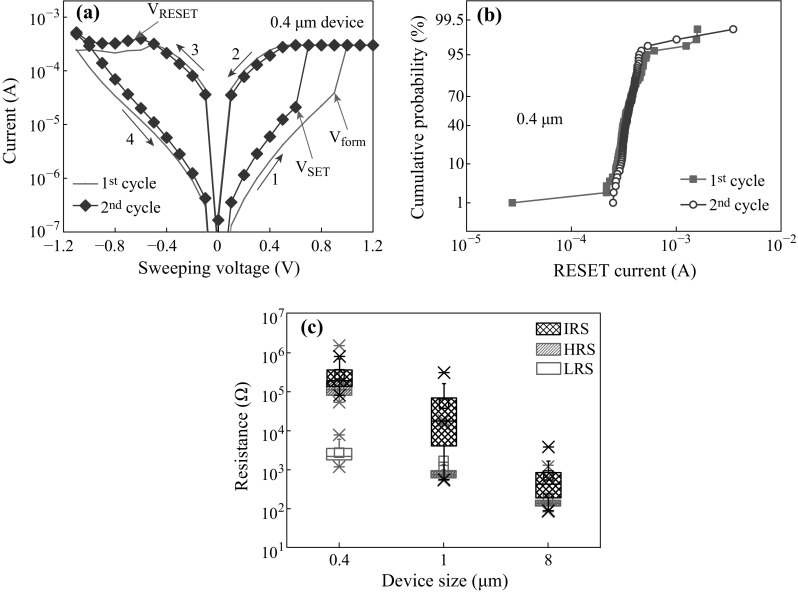
Current–voltage characteristics with device size-dependence switching of 100 measured devices. **a** Bipolar *I*–*V* switching characteristics. **b** Cumulative probability of RESET currents for 0.4-µm device at a low CC of 300 µA, **c** Box chart plot of IRS, HRS, and LRS of 0.4, 1, and 8 µm devices. *Error bars* mean a range of total data distribution

Figure 3a exhibits the current–voltage (I–V) switching characteristics of a 0.4-µm device at a CC of 300 µA. Voltage sweeping direction is shown by arrows 1–4. The device requires a very small forming voltage (*V*_form_) of 0.9 V because of both thin TiO_*x*_ switching layer and vacancy supply from the CrO_*x*_ layer. Small SET (*V*_SET_) and RESET voltages (*V*_RESET_) are found to be 0.6 and −0.6 V, respectively. The RESET currents are found to be 314 and 391 µA for first and second cycle, respectively.

Cumulative probability of RESET currents for the first and second cycles is shown in Fig. 3b. At a 50 % probability, the RESET currents are found to be 329 and 344 µA for 1st and 2nd cycles, respectively. The RESET current is slightly higher (~15 %) than current compliance owing to small current overshoot effect, which can be reduced by optimizing operation current. Further study is needed to evaluate this effect. This current overshoot effect is happening during formation or SET of the devices. Therefore, this structure provides good current clamping and minimizes current overshoot effects, even a one-resistor (1R) configuration.

Cumulative probability of initial resistance state (IRS), high resistance state (HRS), and low resistance state (LRS) for three different device sizes of 0.4, 1, and 8 µm is shown in Fig. 3c. The average values of IRS are 457.5, 46.5, and 670 Ω for the 0.4-, 1-, and 8-µm devices, respectively. The leakage currents increase with increasing device sizes, which are owing to the presence of much higher amount of defects or oxygen vacancies (*V*_o_) in larger device sizes. The average values of HRS/LRS are 144/2.7, 1.5/1.2 kΩ, and 188/175 Ω for 0.4, 1, and 8 µm, respectively at a read voltage (*V*_read_) of 0.2 V. This implies that large size devices (1 and 8 µm) do not show the bipolar resistive switching at a low CC of 300 µA. By reducing the device size as well as leakage current, good resistive switching characteristics could be observed even a simple structure has been designed and fabricated here. In addition, it is found that a non-zero current of approximately 2 × 10^−7^ A is observed at initial and high resistance state, which might possibly be due to capacitive effect. However, a further study is needed.

**Fig. 4:**
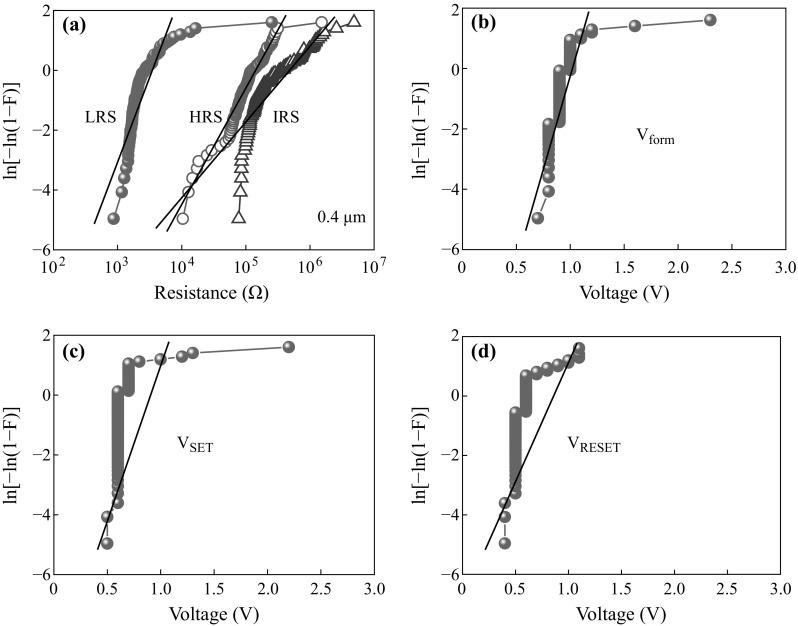
Weibull distribution of **a** IRS, HRS, and LRS, **b** formation, **c** SET, and **d** RESET voltages for 100 arbitrary picked 0.4-µm devices

Figure 4a shows weibull distribution of IRS, HRS ,and LRS for the 0.4-µm devices. These narrow dispersion values interpret that device-to-device uniformity is good with a yield (i.e., switchable devices with consecutive 2 cycles and resistance ratio is >2) of 85 %. We can justify successive switching devices or reliability test as follows by weibull distribution plot [22]. Mathematically, this can be expressed as

1 $$ W\left( Q \right) = \ln \left[ { - \ln \left( {1 - F} \right)} \right], $$

2 $$ F\left( Q \right) = 1 - \exp \left[ { - \left( {\frac{Q}{{\alpha_{63\% } }}} \right)^{\beta } } \right], $$

where *F*(*Q*) is the cumulative distribution function of failure, *Q* is the values of measured data, *β* is the slope value of weibull distribution curve or shape factor which signifies the statistical dispersion of data, and *α*_63 %_ is the scale factor value from weibull distribution at approximately *F* = 63 %. Higher *β* value means that distribution is more uniform. The uniform distribution means that the fitting line should be perpendicular on *X*-axis. Using Eq. (1), weibull distribution patterns of IRS, HRS, LRS, *V*_form_, *V*_SET_, and *V*_RESET_ for the 0.4-µm devices have been depicted in Fig. 4. Using Eq. (2), the values of *β* are found to be from the fitting curves, as shown by straight line. The *β* value of LRS (2.5) distribution is narrower than those of both HRS (1.84) and IRS (1.1), as listed values in Table 1. In addition, IRS distribution of the 0.4-µm devices is narrowest as compared to widely scattered *β* values for 1-µm (0.46) and 8-µm (0.96) devices (not shown here), which may also related to the higher leakage.

**Table 1:** Values of *β* and *α*_63 %_ for the Cr/CrO_*x*_/TiO_*x*_/TiN with a device size of 0.4 µm

Parameters	Slope/shape factor (*β*)	Scale factor (*α*_63 %_)
IRS	1.1	431.5 kΩ
HRS	1.84	139.5 kΩ
LRS	2.5	6 kΩ
*V*_form_	10.4	0.97 V
*V*_SET_	7.34	0.66 V
*V*_REST_	3.9	−0.64 V

It is found that the average values of *V*_form_, *V*_SET_, *V*_RESET_ for the 0.4-µm devices are 0.9, 0.7, and −0.54 V, respectively. As compared to the *V*_SET_, a small *V*_form_ of 0.9 V is necessary to switch a pristine device or it is like a forming-free device, which is very useful for integrated circuit (IC) application and saving extra device process step as well as low cost. The formation step can be avoided by reducing voltage of <3 V then the device will be used directly after fabrication. A 3 V battery can be used directly to program/erase this memory device or extra voltage amplifier which is used in Flash memory is not needed. The *β* values of *V*_form_, *V*_SET_, and *V*_RESET_ are 10.4, 7.34, and 3.9 (Fig. 4b–d), which suggests that formation of the devices is more uniform than those of the SET and RESET voltages. The values of standard deviation of *V*_form_, *V*_SET_, and *V*_RESET_ are 0.174, 0.189, and 0.139, respectively, which assert good device-to-device uniformity yield.

The values of scale factor (*α*_63 %_) for the HRS and LRS are found to be 139.5 and 6 kΩ, respectively. A resistance ratio of approximately 20 is obtained. For a single bit operation, a resistance ratio of >2 is enough to indentify ‘0’ and ‘1’ states [1, 3, 23]. The higher resistance ratio is better for MLC operation. Therefore, scientists are as large as resistance ratio with maintaining other memory parameters, which is also good for future application. The values of *α*_63 %_ for the *V*_form_, *V*_SET_, and *V*_RESET_ are found to be 0.97, 0.66, and −0.64 V, respectively. This suggests that the device could be operated at low voltage of ±1 V.

**Fig. 5:**
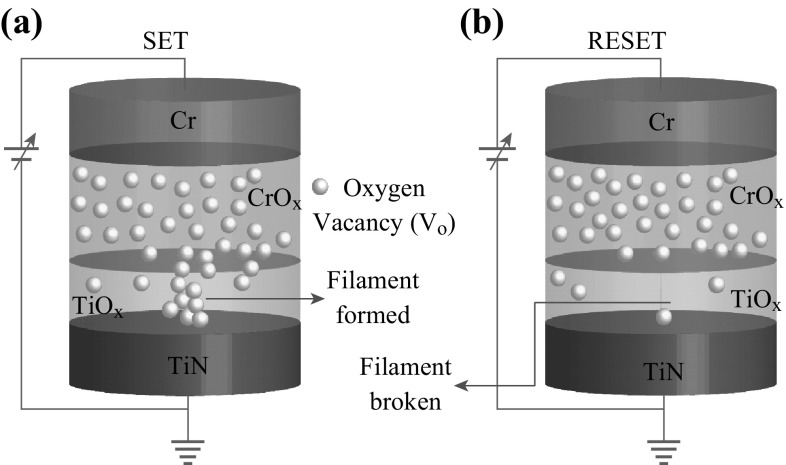
Schematic illustration of switching mechanism. **a** Under SET operation, the oxygen vacancy is migrated from CrO_*x*_ layer to the TiO_*x*_ switching layer and oxygen vacancy conducting filament is formed. **b** Under RESET operation, the oxygen vacancy is moved toward CrO_*x*_ layer from the TiO_*x*_ layer and the filament is ruptured

To evaluate the current conduction mechanism, LRS shows ohmic and HRS shows the space-charge-limited current conduction (SCLC). Stochastic filament formation in TiO_*x*_ layer by oxygen vacancy will lead to respond to the ohmic nature of LRS. On the other hand, injected electrons through the electrodes are exceeded than those thermally generated free electrons in the TiO_*x*_ layer. Therefore, oxygen vacancy filament formation/rupture into the TiO_*x*_ layer under external bias is the switching mechanism, which is also reported by other research groups in different structures [14, 19, 24]. In the Cr/CrO_*x*_/TiO_*x*_/TiN structure, when positive bias is applied on the Cr TE then the potential is distributed in series of TiO_*x*_ and CrO_*x*_ layers. It is true that TiO_*x*_ layer has more insulating property than the CrO_*x*_ layer. Therefore, the potential drop on a pristine device is higher across the TiO_*x*_ layer. Then, Ti–O bonds start to break and electrical conductivity of the TiO_*x*_ layer is increased. In this situation, the potential drop (*V* > *V*_form_ > *V*_SET_) across CrO_*x*_ layer is increased, which results in the oxygen vacancies as a positive charge in the CrO_*x*_ layer moving towards TiO_*x*_ layer as well as the *V*_o_ filament is formed into the TiO_*x*_ layer (Fig. 5a). The device switches from IRS (or HRS) to LRS. By applying negative bias (*V* < *V*_RESET_) on the Cr TE, the oxygen vacancies attracted toward the TE and stored into the CrO_*x*_ layer, which results that the filament is broken (Fig. 5b). Then, the device switches back from LRS to previous HRS. However, there is difference in between IRS and HRS because of Ti–O bonds break during the formation of filament initially. Due to these CrO_*x*_/TiO_*x*_ bilayers’ action, repeatable bipolar resistive switching cycles are observed.

**Fig. 6:**
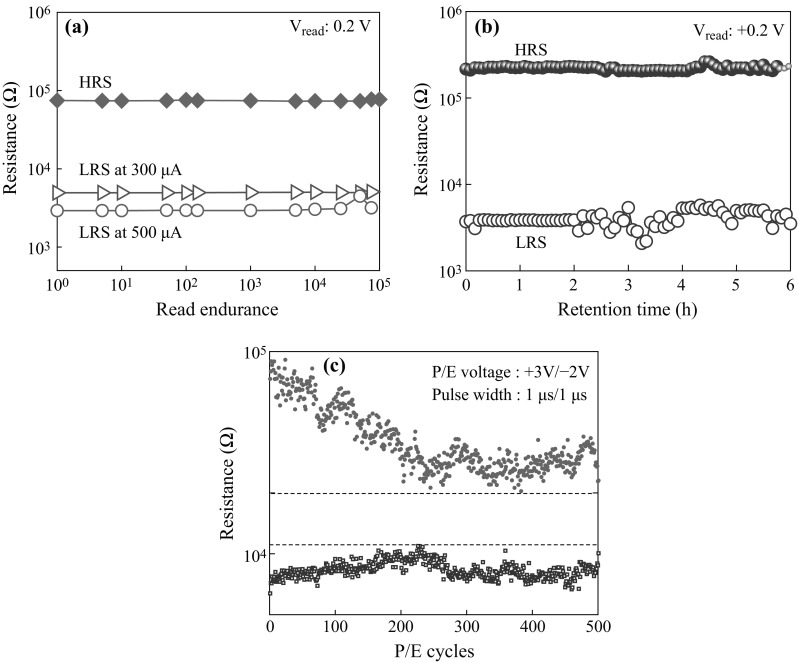
**a** Long read pulse endurance of >10^5^ cycles, **b** Stable data retention of 6 h at a CC of 300 µA, and **c** Good endurance of >500 cycles at a small program/erase pulse width of 1/1 µs obtained for this new Cr/CrO_*x*_/TiO_*x*_/TiN RRAM device

To explore the performance potentiality of the Cr/CrO_*x*_/TiO_*x*_/TiN RRAM devices, program/erase (P/E) endurance and data retention characteristics have been evaluated (Fig. 6). The memory device shows long read pulse endurance measured at *V*_read_ of 0.2 V (Fig. 6a). The device is programed with difference CCs of 300 and 500 µA. After programing the data are read with a pulse width of 500 µs. The LRS values for CCs of 300 and 500 µA are 5 and 3 kΩ, respectively. The value of LRS decreases with increasing current compliance, which can be used as a multi-level cell. After erasing the data are read with a pulse width of 500 µs. The long read pulse endurance of >10^5^ cycles is obtained. Good data retention of 6 h with a good memory window (HRS/LRS) of approximately 40 can be achieved from this RRAM device (Fig. 6b). This memory device shows good P/E endurance of >500 cycles at *V*_read_ of 0.2 V (Fig. 6c). A P/E pulse width of 1/1 µs and voltage of 3/−2 V are applied. A small fluctuation of LRS in both data retention and P/E endurance was observed, which can be assumed to be the generation and redistribution of oxygen vacancies in the TiO_*x*_ switching layer owing to rapid increasing pulse operation. Shen et al. [25] have also discussed about the P/E endurance failure in Pt/BST/SRO RRAM structure due to generation and redistribution of defects in switching material. Further improvement is needed for P/E cycles. Eventually, this memory device with reducing size has very keen potential for future nanoscale non-volatile memory application.

## Conclusions

In summary, observation of resistive switching memory by reducing device size as well as leakage current has been revealed in a new Cr/CrO_*x*_/TiO_*x*_/TiN RRAM device. By measuring 100 random devices it is found that a smaller size device has lower leakage current and resistive switching characteristics are observed. Both TEM image and EDX spectrum confirm a device size of 0.4 × 0.4 µm^2^ with the presence of amorphous TiO_*x*_ and polycrystalline CrO_*x*_ layer is also observed. The 0.4-µm devices can perform resistive switching at a low CC of 300 µA owing to lower leakage current as compared to larger size devices. Weibull plots of IRS, HRS, LRS, *V*_form_, *V*_SET_, and *V*_RESET_ show that more than 85 % devices have good switching with tight distribution. The slope/shape factor indicates the device uniformity. The resistive switching is due to the formation/rupture of oxygen vacancy filament in the TiO_*x*_ switching layer and the CrO_*x*_ layer acts as a vacancy supply layer. Therefore, long read pulse endurance of >10^5^ cycles, data retention of >6 h, and P/E endurance of >500 cycles with a pulse width of 1 µs at a low operation current of 300 µA are obtained. It is concluded that a new Cr/CrO_*x*_/TiO_*x*_/TiN RRAM device has a simple fabrication process and good resistive switching memory characteristics, which will be very promising for future nanoscale non-volatile memory application.
